# A Case of Suprasellar, Intrasellar, and Infrasellar Meningioma Presenting as a Visual Field Defect in an Obese Female

**DOI:** 10.7759/cureus.23071

**Published:** 2022-03-11

**Authors:** Vankadari Venkata Sesha Satya Sagar, Sourya Acharya, Amol Andhale, Sunil Kumar, Dhruv Talwar

**Affiliations:** 1 Department of Medicine, Jawaharlal Nehru Medical College, Datta Meghe Institute of Medical Sciences (Deemed to be University), Wardha, IND

**Keywords:** sella, optic chiasma, visual field defect, hemianopia, meningioma

## Abstract

Bitemporal hemianopia is the most common visual field defect encountered in suprasellar meningiomas compressing the optic chiasma and its vascular supply. It is hard to distinguish between meningioma and tumors that arise from suprasellar, intrasellar, and infrasellar extensions. Clinical findings, hormonal levels, and radiological findings could help in labeling it as meningioma. A 53-year-old obese woman with a history of blurred vision more in the right eye, loss of smell, and a headache was diagnosed with meningioma having suprasellar, intrasellar, and infrasellar extension on neuroimaging. She developed bitemporal hemianopia, which gradually worsened over the course of six months with concomitant headaches and dizziness that was treated with analgesics leading to a delayed diagnosis. As she was symptomatic, it was decided to resect her tumor. The patient underwent an endoscopic transsphenoidal approach for tumor resection. Successful excision of the tumor was accomplished. Postoperatively on further evaluation, the patient’s anosmia and the visual deficit were resolved. This instance shows that meningioma located in the sella can cause symptoms like anosmia and visual field loss, which should not be neglected. It also underlines the significance of visual field evaluation on a regular basis as this might predict radiological and symptomatic progression.

## Introduction

Meningioma is a benign tumor that most commonly arises from the meninges of the brain and spinal cord, accounting for around 37.6% of all and about 50% of all benign brain tumors, with an annual incidence of five cases per 100,000 people. According to the World Health Organization, these tumors are categorized into three grades. A majority of meningiomas are benign and grade 1 in nature. Several predisposing factors are inherited diseases such as neurofibromatosis type 2, radiation exposure, hormone therapy, and family history, which enhance the likelihood of occurrence [1].

The most common locations of meningiomas are skull vault, skull base, parasagittal location, sphenoid ridge, cavernous sinus, olfactory groove, clinoid, petroclival, falcine as well as cerebellopontine angle, foramen magnum, tentorial, and cerebral convexity [1]. Sellar tumors account for 15% of all intracranial tumors. Pituitary adenomas are the most prevalent sellar and suprasellar masses, contributing to about 90% of all cases. Craniopharyngiomas, which can arise anywhere in the infundibulum, are another sellar-based malignancy. Meningiomas themselves constitute only 1% of the sellar masses. Suprasellar/parasellar meningiomas are seen in 5%-10% of all intracranial meningiomas. Meningiomas that arise from the diaphragmatic sellae, also known as the sella turcica, are unusual (1%) [1]. In this case report, the patient has meningioma that is extra-axial evidenced by the dural tail and present in sella and extends up to the suprasellar and infrasellar regions. Visual impairment and a headache are the most frequent presenting complaints.

The clinical presentation of a meningioma is determined by its location and size. As a result, some patients may have no symptoms, while others may have neurological abnormalities. The gold standard radiological investigation for identifying meningioma is brain magnetic resonance imaging (MRI). Asymptomatic and slow-growing meningiomas are usually managed by monitoring and observation. On the other hand, surgery remains the best management choice for fast-growing tumors, big tumors, or symptomatic patients [2].

The majority of meningiomas are sporadic; however, some have been linked to specific disorders and risk factors. Environmental variables such as obesity, alcoholism, ionizing radiation, radiotherapy, hormonal replacement, the use of oral contraceptive pills, and breast cancer can all increase the risk of meningiomas. Suprasellar meningiomas are known for causing visual impairment, which is one of the first and most prevalent symptoms. Due to inferior compression of the optic chiasm, these lesions commonly produce visual field impairments. Bitemporal hemianopsia or superior quadrantanopsia are the visual field deficits that ensue [2].

## Case presentation

A 53-year-old female belonging to a village in central rural India presented with a history of blurred vision more in the right eye for six months. The patient had managed her blurring of vision with limitation such that it resulted in a headache for a duration of two months, which worsened over a period of time and was relieved by analgesics temporarily. The patient also had dizziness and loss of smell for two months. The patient had no history of any comorbidities. On initial assessment, the patient was hemodynamically stable. Her body mass index was 31.4 kg/m^2^. On neurological examination, the patient had anosmia and had a relative afferent pupillary defect on the left eye, which was confirmed by a swinging flashlight test. There was no disc edema. On confrontation, the test patient was found to have a bilateral temporal limitation of the visual field, which was confirmed on perimetry by neuro-ophthalmology unit with a finding of bitemporal hemianopia which raised concern for neuroimaging. Hematological parameters gave no clue as such. Her hormonal profile was also normal apart from marginally raised serum testosterone of 81 ng/dl (normal range in females: 15-70 ng/dL).

**Figure 1:**
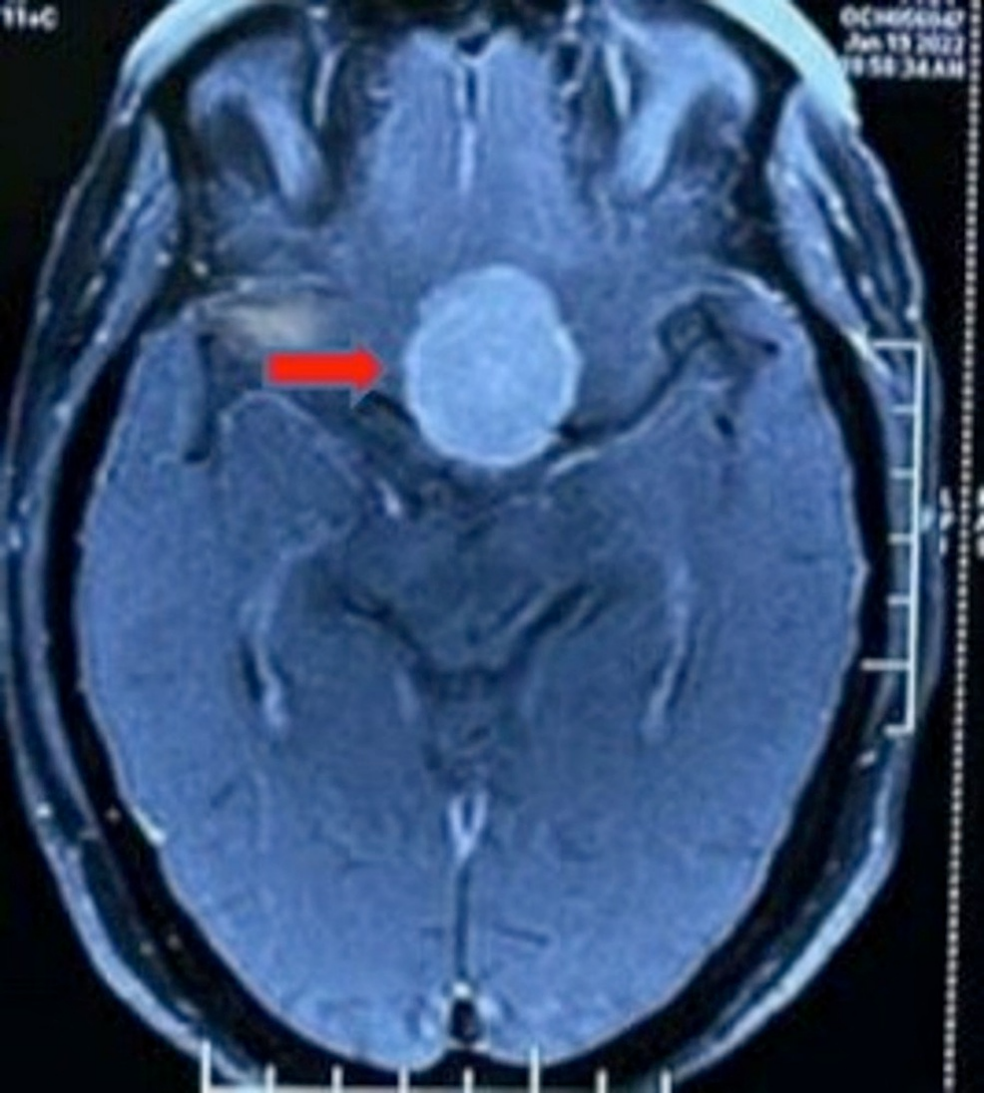
MRI axial section revealed the presence of extra-axial suprasellar, intrasellar, and infrasellar mass lesion of size 4.3 cm x 3.2 cm x 2.9 cm with homogenous post-contrast enhancement. Cerebral edema is seen in the left frontal parenchyma involving gyrus rectus and left frontal white matter. The red arrow indicates the above-mentioned lesion.

**Figure 2:**
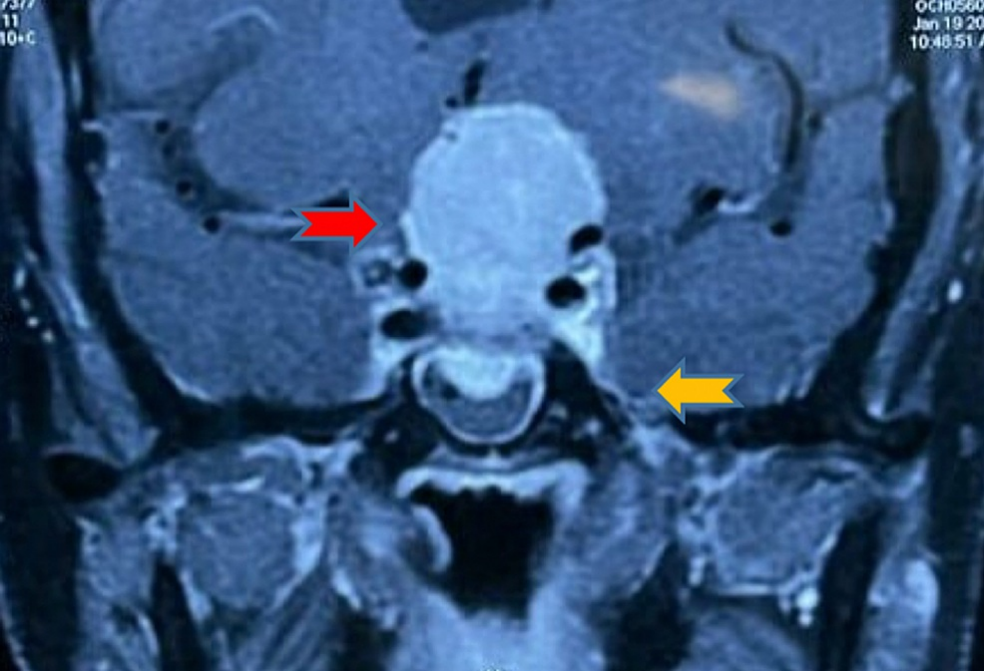
MRI coronal section revealed irregularity of planum sphenoidale with an inferior extension of the lesion in sphenoid sinus with dural enhancement along planum sphenoidale. This image shows the lesion causing compression and posterior displacement of the anterior pituitary gland with maintained fat planes. The red arrow shows meningioma, and the yellow arrow shows the dural tail.

Suspecting pituitary tumor patient's neuroimaging (MRI brain) was performed, which revealed the presence of extra-axial suprasellar, intrasellar, and infrasellar mass lesions of size 4.3 cm x 3.2 cm x 2.9 cm with homogenous post-contrast enhancement as shown in Figure 1. There was an irregularity of planum sphenoidale with the inferior extension of the lesion in sphenoid sinus with dural enhancement along planum sphenoidale. The lesion causing compression and posterior displacement of the anterior pituitary gland with maintained fat planes is shown in Figure 2. Cerebral edema is seen in the left frontal parenchyma involving gyrus rectus and left frontal white matter. Findings are suggestive of meningioma and chronic ischemic foci seen in bilateral periventricular white matter, persistent cavum septum pellucidum, vergae, and multiple arachnoid granulations seen in posterior fossa along the occipital bone. Neurosurgeons took their part and tumor resection was done via a transsphenoidal approach. On further evaluation in the postoperative period, visual field defect and anosmia improved, and the patient was discharged.

## Discussion

Suprasellar meningiomas are benign, well-circumscribed, and slow-growing meningiomas that arise from the tuberculum sellae. They are often asymptomatic until the anterior visual system is damaged, at which point they cause vision loss in one or both eyes. Furthermore, they may encase major blood vessels, jeopardizing the possibility of complete excision [3]. Hardy and Robert described a variant of meningioma with intrasellar location that arises from the inferior portion of diaphragmatic sellae in 1969. Al-Mefty et al. stated in 1985 that diaphragmatic and tuberculum sellae meningiomas were distinct entities [4]. Kinjo et al. classified the diaphragm sellae tumors based on their origin: Type A originates from the upper leaf of the diaphragm sellae anterior to the pituitary stalk, type B tumors from the similar location of type A but posterior to the pituitary stalk, and type C tumors originated from the inferior leaf [5,6].

Hypertonia or clonus, hyperreflexia, positive Babinski and Hoffman symptoms, paresis, or paralysis are common upper motor neuron findings in intracranial meningioma. Paresis or paralysis of the affected contralateral limb can occur in patients with parasagittal and brain convexity meningiomas. Foster Kennedy syndrome is characterized by anosmia, contralateral papilledema, and ipsilateral optic nerve atrophy caused by olfactory groove meningiomas [1,7].

The ocular, oculomotor, trochlear, trigeminal, and abducens nerves can all be affected by cavernous sinus meningioma. Bulbar palsy, cerebellar features, facial palsy, hearing loss, lower cranial nerve palsies, and neck pain are all signs of posterior fossa and foramen magnum meningiomas. Obstructive hydrocephalus can be caused by intraventricular meningioma. Meningiomas of the parasellar, cavernous sinus, and orbit can cause visual loss and proptosis. Sphenoid ridge meningiomas that affect the supraorbital fissure or the cavernous sinus can cause cranial nerve palsies and seizures in patients. Suprasellar or frontal meningiomas can cause mental, cognitive, and behavioral problems in patients [1,8].

Brain MRI with contrast is the best radiological approach for diagnosing meningioma. Extra-axial lesions exhibit a homogeneous enhancement with a characteristic called the dural tail, which can aid in differentiating them from intra-axial lesions [9]. One of the useful markers for distinguishing an extra-axial lesion from an intra-axial lesion is the white matter buckling sign that was demonstrated as an inward compression or buckling of the white matter, gray-white junction preserved even in the presence of edema. Extracerebral fluid collection in extra-axial lesions such as meningioma is usually associated with white matter buckling [10].

Sellar/suprasellar meningiomas can be misdiagnosed as non-hormone-secreting sellar area masses, particularly the non-functioning pituitary adenoma, both clinically and radiologically. They can result in vision loss, visual field problems, hypopituitarism, hyperprolactinemia, or a combination of these symptoms. Sellar/parasellar meningiomas can cause serious intra-operative bleeding in up to one-third of cases due to their extensive vascular supply, adhesion, and invasiveness into neighboring structures. As a result, they sometimes necessitate a unique surgical strategy, which may include pre-operative endovascular embolization of the tumor vasculature. If a reliable pre-operative diagnosis of sellar/suprasellar meningioma was available, such a procedure could be implemented more efficiently [11].

A diaphragm sellae meningioma must be distinguished from a pituitary macroadenoma because they require various surgical methods. Most intrasellar and suprasellar macroadenomas can be reached by the transsphenoidal approach according to Cappabianca et al., but diaphragm sellae meningiomas may necessitate a craniotomy [12]. The transsphenoidal approach, on the other hand, is recommended or should be tried first for all subdiaphragmatic meningiomas, regardless of whether the lesion is pituitary adenoma or meningioma, even if it has a minor suprasellar extension [11]. Intrasellar meningiomas are difficult to distinguish from intrasellar tumors such as pituitary adenoma, pituicytomas, intrasellar germinomas, craniopharyngiomas, aneurysms, and metastases [13].

This case report described a 53-year-old female who resided in a rural area and presented with loss of smell, blurring of vision, and headache. Upon examination, this revealed anosmia with a relative afferent pupillary defect in the left eye and bitemporal hemianopia, which was later diagnosed on neuroimaging as meningioma with intrasellar, suprasellar, and infrasellar extension, which is extra-axially evidenced by the dural tail, and it was decided to resect the tumor by surgical excision via transsphenoidal approach and executed with a successful outcome of improvement in anosmia, visual field, and headache. This delay in the diagnosis for six months after the appearance of symptoms warns of a lack of awareness among the rural population regarding the importance of symptoms like loss of smell and visual abnormalities, which can point toward underlying serious medical illness. Hence, there is a need for increasing awareness, especially in rural and remote areas regarding the importance of such symptoms and the need for accessing healthcare centers for the same.

Another important aspect of our case was the risk factor of obesity in the patient, which might have contributed to the development of meningioma. Previously, in a meta-analysis conducted by Shao et al., it was concluded that obesity was associated with a greater risk of meningioma [14]. Obesity is associated with increased concentration of testosterone in females, which leads to promotion and proliferation of cells along with local production of insulin-like growth factor-1. Estrogen is also associated with an increase in insulin-like growth factor that stimulates the growth of the cell and inhibits apoptosis [15]. As our patient's serum testosterone was marginally raised, obesity could be the hidden culprit behind the development of meningioma.

Although visual rare and atypical presentations of meningioma have been reported before [16,17], this case highlights the importance of simple symptoms such as loss of smell and blurring of vision in cases with certain risk factors. Hence, these symptoms occurring especially in an obese individual should not be overlooked as they can be the tell-tale signs of underlying malignancy, which if resected in time can lead to dramatic improvements in the symptoms while preventing morbidity and mortality.

## Conclusions

A case of meningioma is described, which appears to have begun in the sella and progressed to the suprasellar and infrasellar regions. Depending upon the site of the lesion and compression of vascular or neural structures, various scotomas were identified in the literature such as altitudinal hemianopia; homonymous hemianopia; bitemporal hemianopia; bitemporal upper quadrantic, lower quadrantic, central, and centrocecal scotomas; bitemporal scotoma; etc. This instance shows that meningioma located in sella compresses the optic chiasma and causes bitemporal hemianopia type of visual field loss. Hence, suspecting lesions in the brain and advising neuroimaging are crucial in patients presenting with partial visual field defects. Pituitary hormonal evaluation is mandatory for the management of underlying endocrine disturbances. It also underlines the significance of visual field evaluation on a regular basis as this might predict radiological and symptomatic progression.
